# Bilateral ovarian masses with different histopathology in each ovary

**DOI:** 10.1002/ccr3.1466

**Published:** 2018-03-05

**Authors:** Saeed Baradwan, Haneen Alalyani, Amira Baradwan, Afnan Baradwan, Maram Al‐Ghamdi, Jameel Alnemari, Dania Al‐Jaroudi

**Affiliations:** ^1^ Department of Obstetrics and Gynecology King Fahad Medical City Riyadh Saudi Arabia; ^2^ Faculty of Medicine Princess Nourah Bint Abdulrahman University Riyadh Saudi Arabia; ^3^ Faculty of Medicine King Abdulaziz University Jeddah Saudi Arabia; ^4^ Department of Obstetrics and Gynecology King Abdulaziz University Jeddah Saudi Arabia; ^5^ Department of Radiology King Fahad Medical City Riyadh Saudi Arabia; ^6^ Department of Pathology Prince Mohammed bin Abdulaziz Hospital Riyadh Saudi Arabia; ^7^ Reproductive Endocrine and Infertility Medicine Department King Fahad Medical City Riyadh Saudi Arabia

**Keywords:** Gynecology, mucinous cystadenoma, ovarian cysts, serous cystadenoma

## Abstract

We document the rare occurrence of multiple primary benign lesions that can occur in bilateral ovarian masses with benign imaging appearances and tumor markers. In addition, this case report contributes important information that may aid physicians in guiding their patients to make optimal clinical decisions together.

## Introduction

A female's lifetime risk of having ovarian tumor is 6.0–7.0% 1, and these tumors account for up to 30% of all cancers of the female genital system. Surface epithelial tumors are the most common variety and accounts for approximately 65–75% of all ovarian tumors 2. The most common type of epithelial ovarian neoplasms encountered is benign cystadenomas, of which 75% are serous cystadenomas and 25% are mucinous cystadenomas 3. The occurrence of mixed epithelial tumors is rare, while the occurrence of two different types of ovarian tumors in each of the ovaries is very rare, with only few cases having been documented. We present a case of a 35‐year‐old woman with bilateral ovarian mass treated by laparoscopic cystectomy, and histopathological examination revealed two different ovarian tumors.

## Case Report

A 35‐year‐old nulligravid woman presented to our gynecology outpatient clinic of the King Fahad Medical City, Saudi Arabia, with gradual distension of the abdomen and discomfort over 1 year. The swelling was accompanied by mild lower abdominal pain, constipation, and poor appetite. There was no history of vomiting or other gastrointestinal symptoms, urinary symptoms, colicky pain, and fainting attacks. She had no previous history of any illnesses, allergies, or operations. She denied the use of any medications. There was no family history of malignancies. Her menarche commenced at the age of 12 years.

Her body weight was 80 kg, her height was 161 cm, and her BMI was 30.86 kg/m^2^. Physical examination demonstrated that there was no jaundice, edema, or lymphadenopathy, and secondary sexual characteristics were evident. Abdominal examination revealed a large ill‐defined pelvic‐abdominal cystic mass extending from the pubis up to the umbilicus with an abdominal girth of 95 cm. There was dullness upon percussion but no tenderness. Upon auscultation, the intestinal sounds were normal. Her external genitalia were normal with no abnormality detected by speculum examination. Bimanual examination revealed a normal‐sized uterus, and a cystic mass was felt bilaterally near the posterior fornix that was approximately 7 cm in diameter.

**Figure 1:**
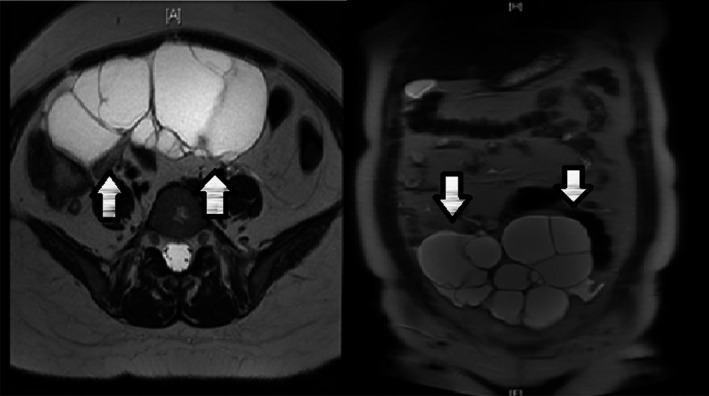
Magnetic resonance imaging (MRI) findings were consistent with bilateral multiloculated cystic ovarian lesions. The right side measures 13.6 × 15.4 × 6.8 cm, and the left side measures 3.6 × 7.4 × 3.1 cm in the anteroposterior, transverse, and craniocaudal dimensions.

Transabdominal and transvaginal ultrasound were performed, which showed a bilateral pelvic multiloculated cystic mass approximately 13 × 10 cm in the right ovary and 6 × 5 cm in the left ovary, with evidence of solid components and septations. The uterus was normal, and endometrial thickness was 8 mm. CA‐125 was 30IU/mL, and other tumor markers (alpha‐fetoprotein, lactate dehydrogenase, carcinoembryonic antigen, beta human chorionic gonadotropin) were within normal ranges. Magnetic resonance imaging (MRI) findings were consistent with bilateral multiloculated cystic ovarian lesions. The cyst on the right side measured 13.6 × 15.4 × 6.8 cm, while that on the left side measured 3.6 × 7.4 × 3.1 cm in the anteroposterior, transverse, and craniocaudal dimensions (Fig. 1). Thus, an ovarian cystadenoma was suspected. No abdominopelvic metastases or lymphadenopathy was reported. After the patient was counseled, she signed informed consent for laparoscopic bilateral ovarian cystectomy. The procedure was performed without complications. Intraoperatively, both ovarian cyst walls were identified and removed using blunt dissection with countertraction without disruption of the capsule. The specimen was intact, placed in the Endo Catch and sent for histopathology.

**Figure 2:**
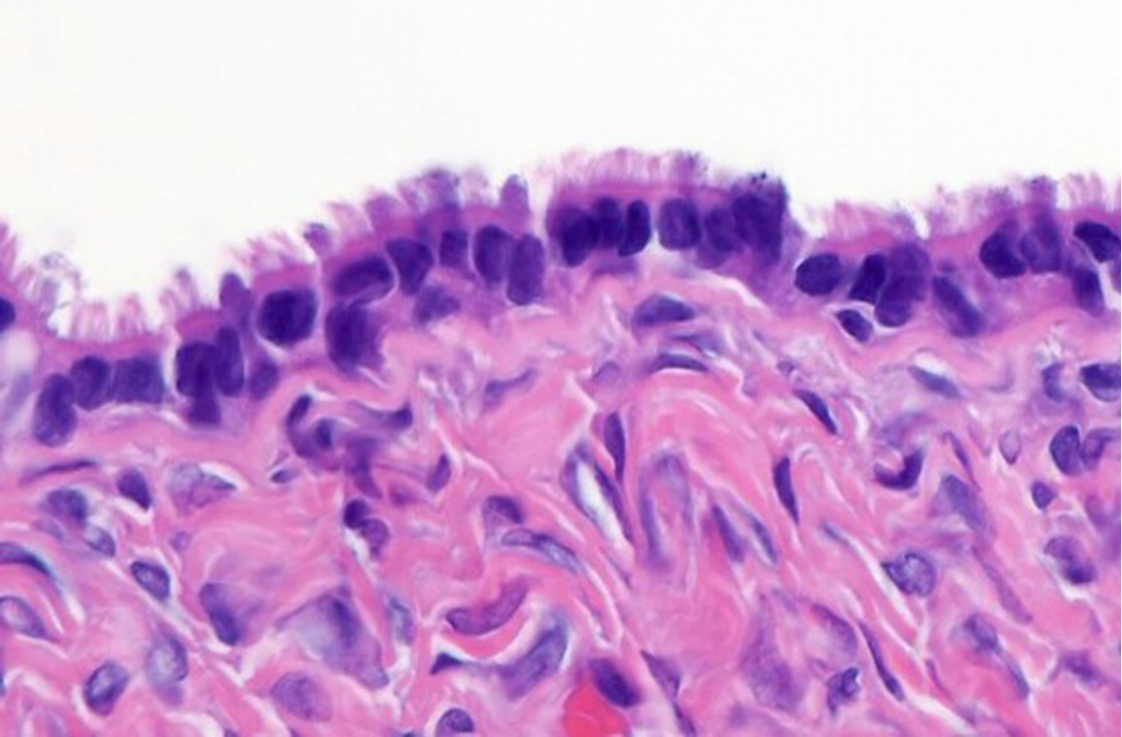
Benign serous tumors of the right ovarian cyst are thin‐walled unilocular cysts that are lined by ciliated pseudostratified cuboidal or columnar epithelium.

**Figure 3:**
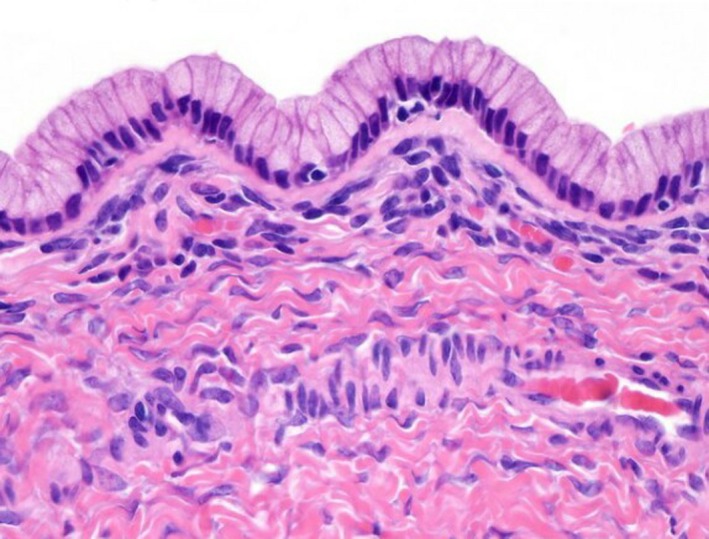
Benign mucinous tumors of the ovary consist of simple, nonstratified columnar epithelium with basally‐located hyperchromatic nuclei and resemble gastric foveolar epithelium.

Histopathological examination revealed that the right cyst was approximately 14 cm with surface papillary excrescences, containing straw‐colored fluid (Fig. 2). The left ovarian cyst was approximately 6 cm and was multiloculated with thick walls. Microscopic examination of the right ovary showed that the cyst wall was lined by simple columnar lining with papillary proliferations, while the left cyst revealed a thick wall with endocervical‐like mucinous cell lining (Fig. 3). The diagnosis was made as right ovarian benign serous cystadenoma and left ovarian benign mucinous cystadenoma.

The postoperative period was uneventful. The patient was discharged on the 2nd postoperative day. She returned back to her normal daily activities and was advised to follow‐up after 4 weeks. Consent for publication of the report was obtained from the patient as well as from the institutional review board (IRB).

## Discussion

Ovarian neoplasms may be divided into four main groups, which include epithelial tumors (65–75%), germ cell tumors (15%), sex‐chord‐stromal tumors (5–10%), and metastatic tumors (10%) 2. The epithelial tumors are among the most prevalent, with the single most common of benign ovarian neoplasm being cystic teratoma. Others have reported serous cystadenoma as the most common type. Serous or mucinous cystadenomas of the ovary arise from the Mullerian germinal epithelium and usually present after puberty 3. Most serous tumors or 50% are benign; however, 15% are borderline, and 35% are invasive carcinomas 4. Mucinous cystadenoma is usually unilateral. This is also the case in benign serous tumors, of which only 20% are bilateral. In this case, we report a rare occurrence of two different types of benign epithelial histopathology in each of the ovaries.

The most common complications of benign ovarian cysts are torsion, hemorrhage, and rupture. Small ovarian cysts are usually asymptomatic and may be found incidentally either clinically or on ultrasound. There are many differential diagnoses for ovarian cysts, such as functional cysts, omental cysts, and mesenteric cysts 5. The most common management options are conservative surgery, ovarian cystectomy, and salpingo‐oophorectomy for benign lesions 6. In young women, one of the main goals is to preserve the reproductive and hormonal functions of the ovaries while preventing recurrence. In this case, we performed treatment with laparoscopic bilateral ovarian cystectomy with the main aim of preserving the patient's hormonal functions.

Microscopic features demonstrate that the serous cystadenoma is lined by a flat (one cell layer) ciliated epithelium covering broad fibrous stromal cores with bland ovoid basal nuclei. On the other hand, microscopic features demonstrate that the mucinous cystadenoma has a layer of columnar cells that are endocervical‐like or intestinal‐like, with uniform round or oval basal nuclei and clear or amphophilic cytoplasm‐lined fibrous stroma 7.

Most of the bilateral ovarian masses with multiple primary cancers in the literature are malignant in nature 8. In our case, both histopathological types were benign, which included serous cystadenoma in one ovary and mucinous cystadenoma in the other ovary. Only a few cases have been reported as bilateral ovarian masses with multiple primary benign lesion 9.

## Conclusion

We document the rare occurrence of multiple primary benign lesions that can occur in bilateral ovarian masses with benign imaging appearance and tumor marker. In addition, this case report contributes important information that may aid physicians in guiding their patients to make optimal clinical decisions together.

## Authorship

SB: literature research, drafting of the manuscript, and corresponding author. HA and AMB: wrote the case description. MA: provided the description of the radiology images. JA: provided the description of the pathology images. AFB: reviewed the literature and involved in writing. DA: critically reviewed and revised the manuscript. All authors read and approved the final manuscript.

## Conflict of Interest

The authors have nothing to disclose and there was no funding for the study. There are no conflict of interests. The work was not presented at any meeting.
